# Epitope Prediction Assays Combined with Validation Assays Strongly Narrows down Putative Cytotoxic T Lymphocyte Epitopes

**DOI:** 10.3390/vaccines3020203

**Published:** 2015-03-24

**Authors:** Peng Peng Ip, Hans W. Nijman, Toos Daemen

**Affiliations:** 1 Department of Medical Microbiology, University Medical Center Groningen, University of Groningen, P.O. Box 30.001, HPC EB88, 9700 RB Groningen, The Netherlands; E-Mails: p.p.ip@umcg.nl (P.P.I); c.a.h.h.daemen@umcg.nl (T.D.); 2 Department of Gynecological Oncology, University Medical Center Groningen, University of Groningen, P.O. Box 30.001, HPC EB88, 9700 RB Groningen, The Netherlands; E-Mail: h.w.nijman@umcg.nl

**Keywords:** HCV, CTL epitopes, Th epitopes, therapeutic vaccine

## Abstract

Tumor vaccine design requires prediction and validation of immunogenic MHC class I epitopes expressed by target cells as well as MHC class II epitopes expressed by antigen-presenting cells essential for the induction of optimal immune responses. Epitope prediction methods are based on different algorithms and are instrumental for a first screening of possible epitopes. However, their results do not reflect a one-to-one correlation with experimental data. We combined several *in silico* prediction methods to unravel the most promising C57BL/6 mouse-restricted Hepatitis C virus (HCV) MHC class I epitopes and validated these epitopes *in vitro* and *in vivo*. Cytotoxic T lymphocyte (CTL) epitopes within the HCV non-structural proteins were identified, and proteasomal cleavage sites and helper T cell (Th) epitopes at close proximity to these CTL epitopes were analyzed using multiple prediction algorithms. This combined *in silico* analysis enhances the precision of identification of functional HCV-specific CTL epitopes. This approach will be applicable to the design of human vaccines not only for HCV, but also for other antigens in which T-cell responses play a crucial role.

## 1. Introduction

Hepatitis C virus (HCV) primarily infects and persists in human hepatocytes. Clearance of HCV involves killing virus-infected cells through cellular immunity, particularly cytotoxic T lymphocytes (CTLs). However, 80% of HCV-infected patients are not able to clear the virus, meaning HCV infection persists. Development of chronic HCV results in liver cirrhosis, and 10% to 15% of chronically infected patients develop hepatocellular carcinoma in 10 to 15 years after the primary infection. During disease progression, viral mutants arise due to the high mutation rate of the HCV genome and immune pressure [1,2].

In 2011 two antiviral HCV protease inhibitors, telaprevir and boceprevir, were approved for treatment for chronic HCV infection next to/combined with the standard treatment with pegylated interferon alpha and ribavirin. Although the sustained antiviral response was improved when using these new drugs, two third of chronically HCV-infected patients are still non-responders to all available treatments [3,4]. Antiviral treatments inhibit HCV replication but do not induce HCV-specific CTLs, which is crucial for clearance of HCV-infected cells [5]. Therefore, to effectively control HCV infection, therapeutic vaccines that aim to induce robust cellular immunity should be developed and used in combination with current HCV therapies.

Spontaneous resolution of HCV infection is positively correlated to the activation of a wide T-cell repertoire recognizing multiple HCV epitopes [6,7]. CTL epitopes can be predicted by mathematical algorithms that compare the protein sequence of interest to a large database of known epitopes. However, for HCV, experimentally verified data on most of these predicted epitopes are limited. Some protective CTL epitopes have been identified in HCV-infected patients [8,9,10]. However, selection of epitopes to be included in a personalized vaccine is crucial and should take into account differences in virus genotypes and subtypes and the presence of escape viral mutants. Thus, to define high-potential candidates for a therapeutic HCV vaccine, it is crucial to identify virus sequences and their corresponding antigenic epitopes using high-throughput methods.

In this study, using a murine model, we aimed to set up a procedure to define and subsequently verify protective HCV CTL epitopes. We selected immunogenic epitopes within conserved HCV proteins based on the results from several mathematical algorithms available. Next, we measured the binding affinity of the predicted CTL peptides to MHC class I molecules *in vitro*. Furthermore, we determined the immunogenicity of the identified epitopes *in vivo*.

## 2. Materials and Methods

### 2.1. Prediction Algorithms and Nucleotide Sequences

HCV peptides associated with MHC class I molecules (H-2D^b^ and H-2K^b^) were predicted with SYFPEITHI, NetMHCpan 2.8 and Immune epitope database and analysis resource (IEDB). HCV peptides associated with MHC class II molecules (H-2-IA^b^) were predicted with IEDB. Proteasomal cleavage sites of the selected HCV peptides were predicted with MAPPP, PAProC I and Netchop. Protein analyzed: nsPs of HCV genotype 1 (H77) (Genomic sequence: NC_004102.1), ovalbumin (Genomic sequence: NC_006089.3) and E7 protein of Human papillomavirus type 16 (Genomic sequence: NC_001526.2).

### 2.2. Synthetic Peptides

Long synthetic peptides (13 to 18-mers) were kindly received from BEI Resources, NIAID, NIH: Peptide Array, Hepatitis C Virus, H77, NS2 protein, NR-3751; NS3 protein, NR-3752; NS4A protein, NR-3753; NS4B protein, NR-3754; NS5A protein, NR-3755; NS5B protein, NR-3756. Short synthetic peptides (8 to 9-mers) were manufactured by the Leiden University Medical Center, the Netherlands. The purities of the synthetic peptide were analyzed with HPLC. All peptides had a purity of >90%.

### 2.3. MHC Class I Stabilization Assay

RMA-S cells were maintained in IMDM (Life Technologies, Bleiswijk, The Netherlands) supplemented with 10% FBS and 100 U/mL penicillin and 100 μg/mL streptomycin (Life Technologies). RMA-S cells were cultured at 26 °C for 48 h to induce expression of MHC class I molecules. Cells were then incubated with various concentrations of synthetic peptides at 26 °C for 4 h, followed by a 1-h culture at 37 °C. Cells were stained with APC-anti-H-2K^b^ Ab (clone: AF6-88.5.5.3) and FITC-anti-H-2D^b^ Ab (clone: 28-14-8) (eBioscience, Vienna, Austria). The surface expression of MHC class I molecules were analyzed by FACSCalibur cytometer (BD Biosciences, Breda, The Netherlands) and data analyzed with FlowJo software (Treestar, Ashland, OR, USA). Fluorescence index was calculated by dividing the median fluorescence intensity (MFI) value of cells incubated with SLPs by the MFI value of cells incubated with equivalent concentration of DMSO.

### 2.4. Mice

Specific pathogen-free female inbred C57BL/6JOlaHsd (H-2^b^) mice expressing both MHC class I K^b^ and D^b^ molecules were obtained from Harlan CPB (Zeist, The Netherlands) and kept under the institute guidelines. Mice were 8 to 10 weeks of age at the start of all experiments. Animal experiments were approved by the Institutional Animal Care and Use Committee of the University Medical Center of Groningen (DEC 5946).

### 2.5. Recombinant Semliki Forest Virus (rSFV) Particles Production and Immunizations

SFV plasmids (pSFVeNS2'-5B', pSFVeNS3/4A and pSFVeNS5A/B') containing HCV non-structure proteins were constructed by sub-cloning the HCV fragments from the full-length cDNA of HCV H77 genotype 1a consensus sequence (H/FL) into pSFVe (pSFV4.2 enclosed with translational enhancer, foot-and-mouth disease virus 2A auto-protease fragment) [11]. H/FL was provided by Charles M Rice via Apath, LLC (AIDS Research and Reference Reagent Program, Division of AIDS, NIAID, NIH: p90HCVconsensuslongpU) [12]. pSFV4.2 was provided by P Liljestrom (Karolinska Institute, Stockholm, Sweden). pSFVeNS2'-5B' (16,838 bps) contains part of the HCV NS2 and NS5B DNA and the complete HCV NS3, NS4A and NS5A DNA (nucleotide position 3106 to 9220 of H/FL). pSFVeNS3/4A (12,839 bps) contains the complete HCV NS3 and NS4A DNA (nucleotide position 3391 to 5543 of H/FL). pSFVeNS5A/B' (13,700 bps) contains the complete HCV NS5A DNA and part of the NS5B DNA (nucleotide position 6258 to 9220 of H/FL). Recombinant SFV particles (rSFVeNS2'-5B', rSFVeNS3/4A and rSFVeNS5A/B') were produced as previously described [11]. In brief, SFV particles were produced by the co-electroporation of BHK-21 cells with an RNA encoding the SFV replicase and the transgene and a helper RNA encoding the structural proteins of SFV. The rSFV replicon particles produced by the transfected BHK-21 cells were purified on a discontinuous sucrose density gradient. rSFV particles were titrated on BHK-21 cells using a polyclonal rabbit anti-replicase (nsP3) antibody (a kind gift from T. Ahola (Biocentre Viiki, Helsinki, Finland)). Mice were primed and boosted immunized intramuscularly with a 2-week interval with 5 × 10^6^ rSFV in 50 μL (25 μL/thigh muscle) under anesthesia (isoflurane/O_2_). For negative controls, PBS was injected intramuscularly.

### 2.6. Degranulation and IFN-γ Staining

Splenocytes isolated from immunized mice were cultured with 10 μg/mL of synthetic peptides in the presence of anti-CD28 Ab (clone: PV-1, Bioceros B.V., Utrecht, The Netherlands), eFluor^®^ 660-anti-CD107a Ab (clone: eBio1D4B) and eFluor^®^ 660-anti-CD107b Ab (clone: eBioABL-93) in a 96-well plate at 37 °C with 5% CO_2_. As a negative control, splenocytes were cultured without peptides but in the presence of anti-CD28 Ab. One hour after culture, brefeldin A (10 μg/mL) was added and the cultures were incubated for another 4 h. Cells were harvested, washed and stained with LIVE/DEAD^®^ fixable violet dead cell stain kit (Life Technologies), followed by surface staining with PE-Cy7-anti-CD8a Ab (clone: 53-6.7) and intracellular staining with PerCP-Cyanine5.5-anti-IFN-γ Ab (clone: XMG1.2). Unless otherwise indicated, antibodies were purchased from eBioscience. FACS analysis was conducted with LSR-II flow cytometer (BD Biosciences), and data was analyzed with FlowJo software (Treestar, Ashland, OR, USA).

## 3. Results and Discussion

### 3.1. Selection and Characteristics of HCV Synthetic Long Peptides that May Contain CTL Epitopes

The MHC class I epitope prediction algorithms SYFPEITHI [13], NetMHCpan 2.8 [14] and Immune Epitope Database and Analysis Resource (IEDB) [15] were used to predict CTL epitopes within HCV nonstructural proteins (nsPs) that bind H-2K^b^/D^b^. The first round of HCV CTL epitope selection was based on predictions from SYFPEITHI (Table 1). Eighteen CTL epitopes that scored higher than 20 in the SYFPEITHI prediction were selected. In addition, NS5B_1–16_ (H-2^d^) [16] and NS5B_46–63_ (multiple human MHC alleles) [17], which are known binders for other MHC alleles, were selected as negative controls. As a result, twenty CTL epitopes were further analyzed with the NetMHCpan 2.8 and IEDB prediction algorithms. The threshold for a strong binder is <0.5% rank in both NetMHCpan 2.8 and IEDB. Seven out of eighteen SYFPEITHI predicted CTL epitopes were predicted by either NetMHCpan 2.8 or IEDB (Table 1). Surprisingly, the negative controls, NS5B_1–16_ and NS5B_46–63_, were predicted as strong binders by both NetMHCpan 2.8 and IEDB.

**Table 1:** Selection of synthetic long peptides containing CTL epitopes from HCV nsPs by prediction algorithms. Sequences of the long synthetic peptides, for which MHC binding affinity was shown in Figure 1, are shown. Strong binders are depicted in bold and highlighted in grey. The cut off score of SYFPEITHI is set at 20; a high score indicates a strong binder. The cut off score of NetMHCpan 2.8 and IEDB is set at 0.5 (% rank); a low score indicates a strong binder.

Protein/Position	Sequence (CTL Epitopes Are Underlined)	MHC I Peptides Prediction						MHC I Stabilization Assay (Figure 1)		
		SYFPEITHI (>20 strong binding)		NetMHCpan 2.8 (<0.5 strong binding)		IEDB (<0.5 strong binding)		Peptide Concentration = 10 μM ^1,2^		Selected for Short Peptides Synthesis (v)
		H-2D^b^	H-2K^b^	H-2D^b^	H-2K^b^	H-2D^b^	H-2K^b^	H-2D^b^	H-2K^b^	
NS3_72-87_	IQMYTNVDQDLVGWPA	**24**	10	0.8	3	2.05	2.25	+	-	
NS3_165-180_	KAVDFIPVENLGTTMR	**30**	8	2	32	2.5	18.5	-	+	
NS3_214-228_	VPAAYAAQGYKVLVL	0	**22**	10	15	24	6.8	++	+	
NS3_323-340_	ATPPGSVTVSHPNIEEVA	**23**	9	**0.08**	8	**0.3**	9.45	+	-	v
NS3_383-400_	ALGINAVAYYRGLDVSVI	0	**22**	32	1.5	19.1	1.15	++	-	
NS3_507-524_	AETTVRLRAYMNTPGLPV	**22**	11	**0.1**	**0.25**	0.7	0.7	+++	-	v
NS3_525-542_	CQDHLEFWEGVFTGLTHI	0	**21**	50	32	13.95	7.9	++	-	
NS3_547-563_	LSQTKQSGENFPYLVAY	**28**	**22**	**0.3**	1.5	**0.4**	1.15	++	-	
NS3_601-618_	RLGAVQNEVTLTHPITKY	**29**	12	**0.08**	32	**0.2**	12.95	+	-	v
NS5A_58-75_	HCGAEITGHVKNGTMRIV	**24**	8	5	32	2.65	36.5	-	-	
NS5A_98-115_	CTPLPAPNYKFALWRVSA	**20**	12	7	32	19	19	-	-	
NS5A_140-157_	CPCQIPSPEFFTELDGVR	**21**	**22**	8	1.5	6.3	**0.5**	-	-	
NS5A_269-284_	ITRVESENKVVILDSF	**24**	7	4	50	5.1	45.5	-	-	
NS5B_1-16_	SMSYSWTGALVTPCAA	13	11	2	**0.03**	5.3	**0.2**	+	+	v
NS5B_46-63_	CQRQKKVTFDRLQVLDSH	15	11	15	**0.05**	27	**0.25**	-	-	v
NS5B_152-169_	GGRKPARLIVFPDLGVRV	15	**22**	32	15	22	12.35	-	++	v
NS5B_249-266_	ARVAIKSLTERLYVGGPL	**22**	12	0.8	3	1.35	2.8	-	-	
NS5B_329-346_	VQEDAASLRAFTEAMTRY	**20**	12	**0.4**	1.5	9.7	13.95	++	+	
NS5B_402-419_	HTPVNSWLGNIIMFAPTL	**21**	8	7	8	12	2.7	-	-	
NS5B_423-439_	MILMTHFFSVLIARDQL	13	**21**	8	**0.17**	9.1	**0.3**	-	-	v

^1^ Fluorescence index of H-2D^b^: 0.2–0.5 +; 0.5–1.5 ++, >1.5 +++; ^2^ Fluorescence index of H-2K^b^: 0.2–2 +; 2–8 ++; >8 +++.

**Figure 1:**
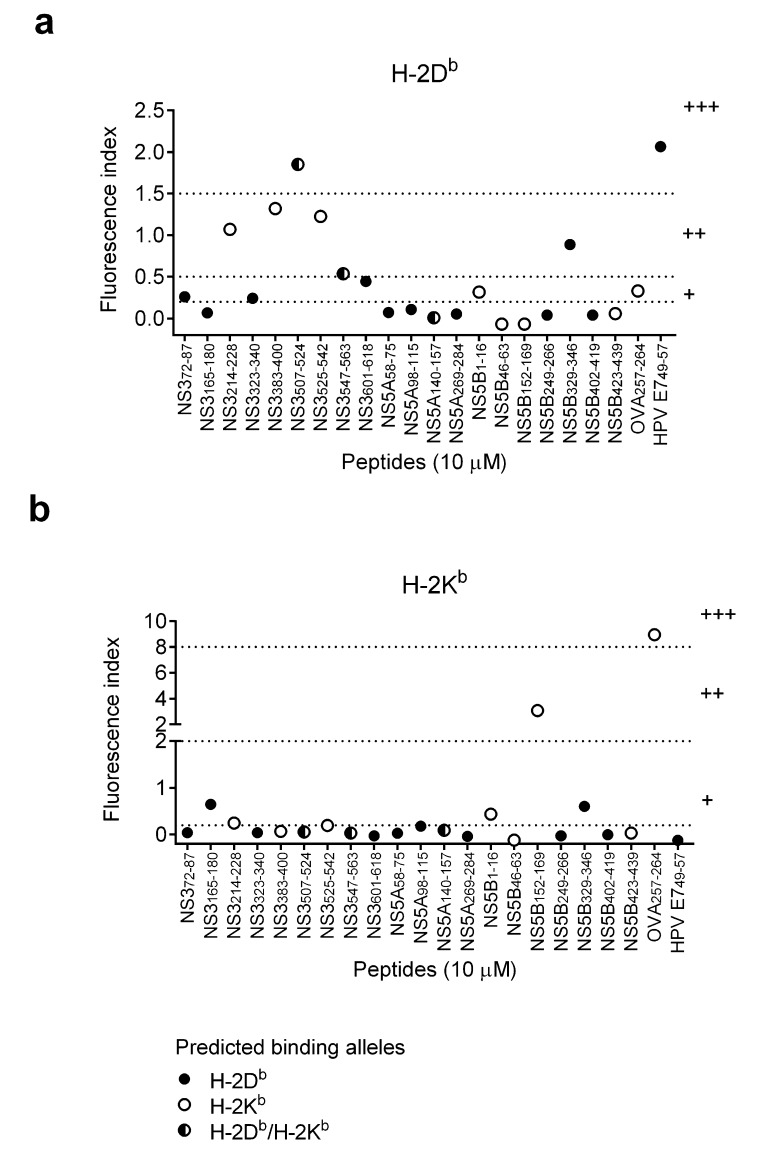
Stabilization of MHC class I molecules with binding of HCV SLPs. To induce MHC class I expression on cell surface, RMA-S cells were cultured at 26 °C for 48 h prior to the incubation with SLPs. Cells were then incubated with 10 μM of SLPs at 26 °C for 4 h, followed by a 1-h cultured at 37 °C. The expression levels of surface MHC class I molecules, (**a**) H-2D^b^ and (**b**) H-2K^b^, were analyzed using flow cytometry. HPV E7_49–57_ and OVA_257–264_ short peptides were positive controls for binding to H-2D^b^ and H-2K^b^ molecules, respectively. Dash lines indicate the cutoff values for H-2D^b^ (0.2–0.5: weak binders (+); 0.5–1.5: intermediate binders (++); >1.5: strong binders (+++)) and H-2K^b^ (0.2–2: weak binders (+); 2–8: intermediate binders (++); >8: strong binders (+++)).

To test the performance of these selected algorithms, the binding capacity of the HCV synthetic long peptides (SLPs) containing the selected CTL epitopes based on the prediction of the three algorithms (Table 1) were determined on MHC class I expressing cells, RMA-S cells (Figure 1). One concentration (10 μM) of SLPs was used in this MHC stabilization assay to screen the possible H-2D^b^ and H-2K^b^ binders. HCV SLPs were classified as weak, intermediate and strong binders according to the ability to stabilize MHC class I molecules on the surface of RMA-S cells. Known CTL epitope peptides of ovalbumin (OVA_257–264_) and human papillomavirus (HPV E7_49–57_) were included as positive controls for H-2K^b^ and H-2D^b^ binding, respectively. Among 13 predicted HCV H-2D^b^ binders, predicted by at least one algorithm (solid and half-open circles), there was one strong binder (NS3_507–524_), two intermediate binders (NS3_547–563_ and NS5B_329–346_) and three weak binders (NS3_72–87_, NS3_323–340_, NS3_601–618_). Three predicted H-2K^b^ binders (open circles) stabilized H-2D^b^ molecules and were considered intermediate binders (NS3_214–228_, NS3_383–400_, NS3_525–542_) (Figure 1a). In ten SLPs that were predicted to be H-2K^b^ binders by at least one algorithm (open and half-open circles), one was classified as an intermediate binder (NS5B_152–169_) and two were weak binders (NS3_214–228_, NS5B_1–16_). Two predicted H-2D^b^ binders (solid circle) stabilized H-2K^b^ molecules and were considered weak binders (NS3_165–180_ and NS5B_329–346_) (Figure 1b).

### 3.2. Binding Affinity of HCV Short Peptides to MHC Class I Molecules

Six out of nine SLPs that contain a CTL epitope predicted by two or more algorithms were able to bind and stabilize MHC class I molecules (Table 1). However, some SLPs that contain CTL epitopes predicted only by SYFPEITHI did not bind to MHC class I molecules. Since SLPs contain extra amino acid flanking the predicted CTL epitopes, binding affinity of SLPs to MHC class I molecules may be reduced [18,19]. Thus, binding capacity of the CTL epitopes was confirmed with short synthetic peptides that contain only the CTL epitope. Short synthetic peptides were selected based on one of the following criteria: (i) CTL epitope has to be predicted by at least 2 algorithms and/or classified as a weak, intermediate or strong binder from the result of the MHC I stabilization assay with SLPs (NS3_323–340_, NS3_507–524_, NS3_601–618_, NS5B_1–16_, NS5B_46–63_, NS5B_423–439_); (ii) CTL epitope has to be predicted by at least 1 algorithm and classified as intermediate or strong binder from the result of MHC I stabilization assay (NS5B_152–169_). Since the accuracy of prediction greatly increased when peptides were analyzed with two or more algorithms, four extra CTL epitopes, which were predicted by at least two algorithms as strong binders, were selected (NS2_139–147_, NS3_265–273_, NS4B_38–46_ and NS5A_280–287_) (Table 2). And also for this analysis, the known CTL epitopes of ovalbumin (OVA_257–264_) and human papillomavirus (HPV E7_49–57_), which are strong H-2K^b^ and H-2D^b^ binders, respectively, were selected as positive controls.

**Figure 2:**
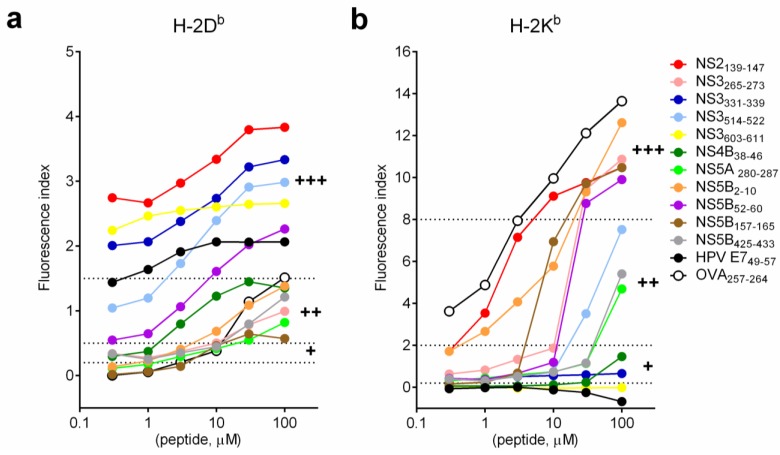
Binding affinity of HCV short peptides to MHC class I molecules. Short synthetic HCV peptides were serial diluted and incubated with RMA-S cells as described in Figure 1. (**a**) H-2D^b^; (**b)** H-2K^b^. Dash lines indicate the cutoff values for H-2D^b^ (0.2–0.5: weak binders (+); 0.5–1.5: intermediate binders (++); >1.5: strong binders (+++)) and H-2K^b^ (0.2–2: weak binders (+); 2–8: intermediate binders (++); >8: strong binders (+++)).

Binding affinity of selected short synthetic peptides on MHC class I molecules was determined by MHC I stabilization assay with various concentrations of peptide (Figure 2). As expected, stabilization of both H-2D^b^ and H-2K^b^ molecules increased with increasing concentration of peptide. The kinetic of the stabilization of H-2D^b^ molecules was similar for all selected short peptides. The plateau of stabilization was reached at approximately 30 μM of peptides (Figure 2a). Two kinetic patterns of stabilizations of H-2K^b^ molecules were observed. For strong binders (at 100 μM, FI > 8; OVA_257–264_, NS5B_2–10_, NS3_265–273_, NS5B_157–165_, NS2_139–147_ and NS5B_52–60_), there was a dose dependent increase of FI and the plateau of stabilization was not detectable even at high peptide concentration (100 μM). For intermediate binders (NS3_514–522_, NS5B_425–433_ and NS5A_280–287_), stabilization of H-2K^b^ molecules was modest at low peptide concentration (0.3–10 μM). However, the stabilization effect increased exponentially once the concentration was above 30 μM. This observation may explain why we did not see strong H-2K^b^ binders when SLPs at 10 μM were used in the MHC I stabilization assay (Figure 1). Results of stabilization of MHC class I molecules with short peptides matched the result from all of the MHC class I prediction algorithms used (H-2D^b^: >90% matched; H-2K^b^: 100% matched) (Table 2).

### 3.3. The Presence of Proteasomal Degradation Sites at the Carboxyterminal Site of the Predicted CTL Epitopes

Under physiological conditions, proteins have to be cleaved by proteasomes into short peptides in order to be loaded onto MHC class I molecules. Proteasomal cleavage sites were predicted with MAPPP, PAProC I and Netchop. MAPPP and Netchop predict the cleavage site of constitutive proteasomes and PAProC I predicts cleavage site of both constitutive proteasomes and immunoproteasomes. From the result of at least two prediction algorithms, proteasomal cleavage sites were present at all strong MHC class I binders (+++) (Table 2).

### 3.4. The Presence of MHC Class II Epitopes Flanking the Predicted CTL Epitopes

It has been observed that immunodominant CTL epitopes such as Influenza B nucleoprotein are flanked and/or overlap with Th epitopes [20,21,22]. Association of CTL and Th epitopes may indeed enhance the immunogenicity of the CTL epitopes *in vivo* [23]. To investigate the presence of MHC class II epitopes flanking the strong MHC class I epitopes, we next performed prediction of MHC class II epitopes with IEDB. Most of the identified high affinity MHC class I epitopes were flanked with at least one predicted MHC class II epitope. Also, two MHC class I epitopes (both H-2D^b^ and H-2K^b^), overlap with a predicted MHC class II epitope (IEDB rank < 10) (NS3_514–522_ and NS5B_2–10_). Flanking of MHC class I epitopes with predicted MHC class II epitopes was also observed in the positive control, OVA_257–264_ (Table 2).

### 3.5. Induction of Peptide-Specific Effector CD8^+^ T Cells in Vivo

**Figure 3:**
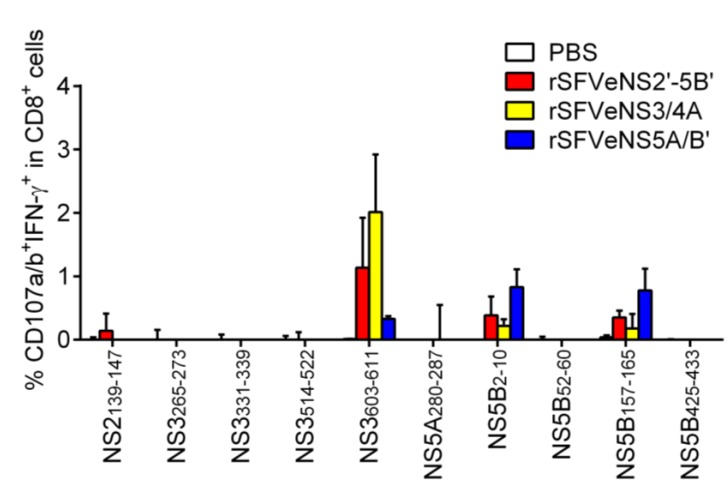
Induction of peptide-specific effector CD8^+^ T cells *in vivo*. Mice were intramuscularly immunized thrice with rSFV expressing HCV nsPs (rSFVeNS2'-5B', rSFVeNS3/4A or rSFVeNS5A/B') or PBS control with a one-week interval. Mice were sacrificed 1 to 3 weeks after the boost immunization. Splenocytes were isolated and cultured with 10 μg/mL of peptides for 5 h before surface and intracellular staining. Background (splenocytes incubated with an equivalent concentration of DMSO) subtraction was applied and values above background are shown. Data represent combined results from three independent experiments, showing the mean +SEM (3–5 mice per group).

Induction of *in vivo* T-cell response depends on the presentation of peptides and the availability, avidity and affinity of precursor CD8^+^ T cells [24,25]. Here, we investigated the induction of T-cell response against the predicted CTL epitopes in mice immunized three times with the rSFV particles expressing all HCV nsPs, NS3/4A or NS5A/B' (rSFVeNS2'-5B', rSFVeNS3/4A or rSFVeNS5A/B'). Splenocytes were isolated 1 to 3 weeks after the last immunization and re-stimulated with selected short peptides in order to induce degranulation (surface expression of CD107a/b) and secretion of IFN-γ by peptide-specific CD8^+^ T cells (Figure 3). rSFV immunizations induced functional effector CD8^+^ T cells (CD107a/b^+^IFN-γ^+^) against NS2_139–147_, NS3_603–611_, NS5B_2–10_ and NS5B_157–165_. When no peptides were added to the splenocytes, we already observed the presence of endogenous CD107a/b^+^IFN-γ^+^CD8^+^ cells from mice immunized with any rSFV particles but not from mice immunized with PBS. This indicates that rSFV immunizations in general induced functional CD8^+^ T-cell responses. To check for a specific response against peptide, background (without peptide re-stimulation) subtraction was applied. Frequencies above zero indicate specific response against the re-stimulating peptide. CD8^+^ T-cell response against NS3_603–611_ [26] was the highest among all selected peptides and was detected in mice immunized with rSFVeNS2'-5B' or rSFVeNS3/4A. Responses against NS5B_2–10_ and NS5B_157–165_ were low and induced in mice immunized with rSFVeNS5A/B' or rSFVeNS2'-5B'. A very low response against NS2_139–147_ was observed only in mice immunized with rSFVeNS2'-5B', not in mice with other immunizations. Of note, all responding peptides were predicted by at least one algorithm for MHC class I prediction. Proteasomal cleavages sites were presented in the carboxyterminals of all four peptides. Three peptides (NS2_139–147_, NS3_603–611_ and NS5B_2–10_) contained predicted MHC class II epitopes in close proximity to their CTL epitopes with relatively high rankings (Table 2).

**Table 2:** Detailed analysis of selected short synthetic HCV peptides. Prediction of proteasomal cleavage sites and MHC class II epitopes flanking the selected CTL epitopes for (**a**) H-2D^b^, (**b**) H-2K^b^. The CTL epitope and its flanking amino acids (11 amino acid at 5' end and 11 amino acid at 3' end of the CTL epitope) were analyzed with the MHC class II prediction algorithm (IEDB). Rankings below 10 were considered strong binders. The values represent the ranking range for the analyzed amino acids. Data were sorted according to the binding affinity of short peptides to RMA-S cells (Figure 2), strong binders at the top of the row (**a**: H-2D^b^; **b**: H-2K^b^). N.D. not determined. Strong binders are depicted in bold and highlighted in grey.

**a**		**MHC I Stabilization Assay ^1^**	**MHC Class I Prediction**			**Proteasomal Cleavage**			**MHC Class II Prediction**		
			**SYFPEITHI (>20 strong binding)**	**NetMHCpan 2.8 (<0.5 strong binding)**	**IEDB (<0.5 strong binding)**	**MAPPP (Cleavage Probability ^3^)**	**PAProC I (Score ^4^)**	**Netchop (Cleavage Probability ^3^)**	**IEDB- H-2-IA^b^ (<10 strong binding)**		
**Protein/Position**	**Sequence**	**H-2D^b^**	**H-2D^b^**	**H-2D^b^**	**H-2D^b^**				**5' of CTL Epitope**	**Complete CTL Epitope**	**3' of CTL Epitope**
NS2_139-147_	YVYNHLTPL	+++	13	**0.15**	0.7	1	0-	0.95	31.64–58.25	**1.82–3.90**	27.51–64.83
NS3_331-339_	VSHPNIEEV	+++	**23**	**0.08**	**0.3**	0.7502	121+++	0.97	**2.21**–12.13	10.72–48.25	31.02–54.21
NS3_514-522_	RAYMNTPGL	+++	**22**	**0.1**	0.7	0	78++	0.95	**5.17**–73.56	**5.97**–22.88	16.57–87.59
NS3_603-611_	GAVQNEVTL	+++	**29**	**0.08**	**0.2**	0	178+++	0.88	**9.35**–55.70	18.51–32.13	16.70–51.34
HPV E7_49-57_	RAHYNIVTF	+++	**23**	**0.08**	**0.2**	1	N.D.	0.91	33.38–53.38	56.31–79.26	69.56–86.81
NS5B_52-60_	VTFDRLQVL	+++	15	15	27	0.5976	0-	0.96	10.39–86.63	71.97–79.66	28.42–85.75
OVA_257-264_	SIINFEKL	++	0	4	**0.2**	1	N.D.	0.97	51.45–69.97	51.88–81.68	**9.57**–82.66
NS4B_38-46_	AVQTNWQKL	++	**23**	**1**	1.9	0	0-	0.72	12.81–32.62	23.98–74.51	21.59–58.54
NS5B_2-10_	MSYSWTGAL	++	13	2	5.3	1	0-	0.87	N.D.	**0.93–0.95**	**2.62**–56.47
NS5B_425-433_	LMTHFFSVL	++	13	8	9.1	0.5009	40++	0.96	11.68–61.33	36.91–72.20	46.08–77.76
NS3_265-273_	ITYSTYGKF	++	9	32	14.3	1	0-	0.209	10.30–47.29	36.17–47.83	49.48–84.06
NS5A_280-287_	ILDSFDPL	++	0	15	**0.2**	0.906	0-	0.92	51.43–86.16	18.50–86.11	20.39–86.16
NS5B_157-165_	ARLIVFPDL	++	15	32	22	0.6886	0-	0.96	44.16–67.37	47.47–56.37	51.99–81.87
**b**		**MHC I Stabilization Assay ^2^**	**MHC Class I Prediction**			**Proteasomal Cleavage**			**MHC Class II Prediction**		
			**SYFPEITHI (>20 strong binding)**	**NetMHCpan 2.8 (<0.5 strong binding)**	**IEDB (<0.5 strong binding)**	**MAPPP (Cleavage Probability ^3^)**	**PAProC I (Score ^4^)**	**Netchop (Cleavage Probability ^3^)**	**IEDB- H-2-IA^b^ (<10 strong binding)**		
**Protein/Position**	**Sequence**	**H-2K^b^**	**H-2K^b^**	**H-2K^b^**	**H-2K^b^**				**5' of CTL Epitope**	**Complete CTL Epitope**	**3' of CTL Epitope**
OVA_257-264_	SIINFEKL	+++	**25**	1.5	**0.35**	1	N.D.	0.97	51.45–69.97	51.88–81.68	**9.57**–82.66
NS5B_2-10_	MSYSWTGAL	+++	11	**0.03**	**0.2**	1	0-	0.87	N.D.	**0.93**–**0.95**	**2.62**–56.47
NS3_265-273_	ITYSTYGKF	+++	11	**0.4**	**0.3**	1	0-	0.209	10.30–47.29	36.17–47.83	49.48–84.06
NS5B_157-165_	ARLIVFPDL	+++	**22**	15	12.35	0.6886	0-	0.96	44.16–67.37	47.47–56.37	51.99–81.87
NS2_139-147_	YVYNHLTPL	+++	11	**0.01**	**0.2**	1	0-	0.95	31.64–58.25	**1.82**–**3.90**	27.51–64.83
NS5B_52-60_	VTFDRLQVL	+++	11	**0.05**	**0.25**	0.5976	0-	0.96	10.39–86.63	71.97–79.66	28.42–85.75
NS3_514-522_	RAYMNTPGL	++	11	**0.25**	0.7	0	78++	0.95	**5.17**–73.56	**5.97**–22.88	16.57 – 87.59
NS5B_425-433_	LMTHFFSVL	++	**21**	**0.17**	**0.3**	0.5009	40++	0.96	11.68–61.33	36.91–72.20	46.08–77.76
NS5A_280-287_	ILDSFDPL	++	**21**	6	1.45	0.906	0-	0.92	51.43–86.16	18.50–86.11	20.39–86.16
NS4B_38-46_	AVQTNWQKL	+	11	15	8.2	0	0-	0.72	12.81–32.62	23.98–74.51	21.59–58.54
NS3_331-339_	VSHPNIEEV	+	9	8	9.45	0.7502	121+++	0.97	**2.21**–12.13	10.72–48.25	31.02–54.21
NS3_603-611_	GAVQNEVTL	-	12	32	12.95	0	178+++	0.88	**9.35**–55.70	18.51–32.13	16.70–51.34
HPV E7_49-57_	RAHYNIVTF	-	8	5	11.2	1	N.D.	0.91	33.38–53.38	56.31–79.26	69.56–86.81

^1^ Fluorescence index of H-2D^b^: 0.2–0.5 +; 0.5–1.5 ++; >1.5 +++; ^2^ Fluorescence index of H-2K^b^: 0.2–2 +; 2–8 ++; >8 +++; ^3^ Cleavage probability: 1 = 100% cleavage; ^4^ PAProC I score: high score indicates high chance for cleavage, also depicted by the number of +.

## 4. Discussion

Identification of protective HCV CTL epitopes is essential in the development of vaccines for patients with chronic HCV infection. Treatment can be personalized based on the circulating HCV genotype(s). Ideally one should identify HCV epitopes expressed by the patients’ infected cells and induce T-cell responses against the identified epitopes with vaccine immunizations. Identification of HCV epitopes from cell lines expressing HLA-A2 and HCV proteins has been performed with electro-spray ionization quadrupole time-of-flight mass spectrometry/tandem mass spectrometry (ESI Q-TOF-MS/MS) and the data were consistent with the results predicted from mathematical algorithms [27]. This method could also be used to identify naturally processed and presented HCV peptides from HCV-infected hepatocytes. However, widespread use of this analysis is limited, as liver biopsies are required for the analysis and taking biopsies is not standard procedure for these patients. Moreover, the percentage of HCV-infected hepatocytes in biopsies may be too low for MS analysis [28,29]. An alternative method is to identify CTL epitopes *in silico* based on the HLA phenotype and the viral sequence identified from blood of HCV-infected patients.

Here we show that the prediction accuracy for the identification of potential CTL epitopes is improved by combining several algorithms for MHC class I epitope and proteasomal cleavage site prediction, and possibly even MHC class II epitope prediction. We narrowed down all HCV nsPs to 22 CTL epitopes using prediction algorithms, of which 10 were proven to bind to H-2^b^ molecules on RMA-S cells. We next showed that immunization with rSFV expressing all nsPs induced a strong epitope-specific T-cell response against one out of ten H-2^b^ binders and a weak response against three epitopes.

Computational analyses used in this study are machine-learning algorithms, *i.e.*, the programs were trained with large databases for reliable results. Therefore, most algorithms favor the detection of immunodominant epitopes. Predictions were made with allele-specific motif methods based on amino acid sequences [30,31]. For example, peptide positions 2 and 9 are classical anchor residues most important for binding to the HLA-A*0201 allele and are typically occupied by leucine, valine and isoleucine [32]. With allele-specific motif methods, peptides containing appropriate amino acids at anchoring positions are predicted to be MHC class I binders, but this type of prediction does not necessarily correlate with the physiological function of the epitopes.

Antigen processing involves a complex regulation of antigen degradation, interaction with chaperones, binding to the peptide-loading complex, loading onto MHC class I molecules and transport to the cell surface [33]. For both presentation of intracellular antigens and cross-presentation of extracellular antigens, antigen degradation by constitutive and/or immune proteasome is required. Upon exposure of IFN-γ in lymphoid cells, the constitutive proteasomes are replaced by immunoproteasomes which have altered preferences for cleavage sites [34]. The immunoproteasomes favor the processing of immunodominant peptides [35]. Normal human liver cells also express immunoproteasomes, as well as constitutive and intermediate proteasomes that enable the broadening of the repertoire of antigenic peptides [36]. To define the physiological function of predicted CTL epitopes, we analyzed the proteasomal cleavage sites recognized by constitutive proteasomes (MAPPP, PAProC I and Netchop) or immunoproteasomes (PAProC I) present at the carboxyterminals of the predicted CTL epitopes. Combining MHC I prediction with proteasomal cleavage prediction indeed increases the accuracy of MHC I peptide prediction [37]. In this study, all predicted strong H-2D^b^ and H-2K^b^ binders contained proteasomal cleavage sites predicted by at least two algorithms.

Activation of CD8^+^ T cells relies on both recognition of MHC I-peptide complexes on APCs and co-stimulatory activation signals provided by adjacent cells such as APCs and/or CD4^+^ helper T cells. Activated CD4^+^ helper T cells orchestrate the activation of CD8^+^ T cells through secretion of IL-2 and/or activation of dendritic cells for up-regulation of MHC I molecules and antigen presentation [23]. Interestingly, some known immunodominant CD8^+^ T cell epitopes such as OVA_257–264_ contain predicted high-affinity Th epitopes that overlap or are in close proximity to one another [23,38]. This may allow presentation of both MHC I and MHC II peptides by the same dendritic cells, resulting in activation of both CD8^+^ and CD4^+^ T cells simultaneously [39]. Alternatively, activated helper T cells acquire MHC II-peptide complexes from APCs as well as co-stimulating molecules, leading to activation of Th1 responses and central memory CD8^+^ T-cells responses [40,41,42]. It is proposed that the uptake of MHC II peptides may be further processed into MHC I peptides which can be loaded onto recycled MHC I molecules and presented to CD8^+^ T cells [23,43,44]. This will lead to higher activation of CD8^+^ T cells as helper T cells provide both antigen stimulation and co-stimulation [45]. Peptides recognized by splenocytes from rSFV-immunized mice (NS2_139–147_ and NS5B_2–10_) overlapped with strong predicted Th epitopes (Table 2). The immunodominant epitope (NS3_603–611_) contains intermediate predicted Th epitopes at the 5' ends of the CTL epitope. Remarkably, MHC class II epitope prediction by IEDB may not identify all possible Th epitopes, since, for example, it has been shown that E7_44–62_, binds to MHC class II molecules *in vitro* but was not predicted as a strong binder [46]. As MHC class II binding sites are abundantly present, however, further studies are needed to prove or reject the hypothesis that there is a correlation between the immunogenicity of class I epitopes and overlap with high-affinity MHC class II epitopes. Besides the results of prediction algorithm, the presence of Th epitopes should be determined using *in vitro* experiments such as an MHC class II binding assay. For the design of vaccines expressing specific CTL epitopes, this could be considered to include Th epitopes that overlap or are in close proximity with CTL epitopes to enhance immune responses. In a separate study, we demonstrated that inclusion of strong universal helper-epitopes in an SFV replicon vaccine expressing human papillomavirus (HPV) antigens strongly enhanced the frequency of HPV specific CTLs [47].

In this study, we identified 1 out of 22 predicted CTL epitopes inducing strong CD8^+^ T-cell responses (NS3_603–611_) and 3 out of 22 inducing minor CD8^+^ T-cell responses (NS2_139–147_, NS5B_2–10_ and NS5B_157–165_). Subdominant CTL epitopes could not be identified with rSFVeNS2'-5B', rSFVeNS3/4A or rSFVeNS5A/B' immunizations. Inclusion of more epitopes may increase competition between T cells of different specificity, resulting in a reduced response to subdominant epitopes [48]. Furthermore, expression of full proteins may reduce the expression level of one particular peptide compared to expression of only one peptide. This may lead to insufficient antigen presentation, resulting in unstable contact between CD8^+^ T cells and APC and thus inability to fully activate T cells [49]. Although the biological function of responses against subdominant epitopes is unknown, an alternative approach to checking the accuracy of *in silico* predictions is to immunize with synthetic long peptides expressing only one particular CTL epitope and its flanking sequences. In this way, the immune response will be directed to that specific epitope.

## 5. Conclusions

To conclude, we validated *in silico* approaches to epitope prediction *in vivo*. We showed that responses against predicted dominant CTL epitopes were induced in mice immunized with HCV-nsPs expressing rSFV. *In vivo* relevance of subdominant epitopes, which were predicted by mathematical algorithms, may have to be confirmed with peptide immunizations. Combining epitope prediction algorithms with proteasomal cleavage site algorithms increases the accuracy of CTL epitope identification. The predictive value of strong Th epitopes overlapping with, or in close proximity to, CTL epitopes remains to be established. Thus, potential CTL with or without Th epitopes as candidates for therapeutic human vaccine development can be identified and narrowed down. For clinical practice, the establishment of an *ex vivo* stimulation assay with patients’ PBMCs is warranted to select the rationally designed candidate vaccine that is most immunogenic for that specific patient.
